# The Effects of Alcohol Consumption on Cardiometabolic Health Outcomes Following Weight Loss in Premenopausal Women with Obesity: A Pilot Randomized Controlled Trial

**DOI:** 10.3390/ijerph17155302

**Published:** 2020-07-23

**Authors:** John W. Apolzan, Robbie A. Beyl, Corby K. Martin, Frank L. Greenway, Ursula White

**Affiliations:** Pennington Biomedical Research Center, Louisiana State University System, Baton Rouge, LA 70808, USA; Robbie.Beyl@pbrc.edu (R.A.B.); Corby.Martin@pbrc.edu (C.K.M.); Frank.Greenway@pbrc.edu (F.L.G.)

**Keywords:** ethanol, alcohol, weight loss, body weight, obesity, triglycerides, visceral fat, cholesterol, uric acid, blood pressure

## Abstract

Alcohol (i.e., ethanol) is consumed regularly by much of the adult population; yet, the health effects associated with its use are not well-characterized. Clinical interventions to investigate the effects of moderate alcohol consumption on metabolic outcomes, including adiposity and cardiovascular risk factors, are limited and have yielded conflicting data. In addition, no study has reported the effects of routine alcohol intake during weight loss in a controlled feeding trial. We present the first randomized controlled pilot trial to investigate the effects of moderate alcohol consumption on metabolic outcomes during weight loss in women with obesity. Both groups consumed 30% energy restricted diets and were randomized to either an ethanol-free control (CTL) group or a group (EtOH) that consumed 35 g ethanol daily for eight weeks. Our findings demonstrate that, despite similar weight loss, the decrease in mean arterial pressure was attenuated in the EtOH group, relative to the CTL group (*p* = 0.02). In addition, decreases in other outcomes, including visceral adipose tissue (*p* = 0.23), circulating lipids (triglycerides (*p* = 0.11) and cholesterol (*p* = 0.11)), and uric acid (*p* = 0.07) tended to be attenuated with alcohol consumption. These pilot data provide potential evidence that moderate alcohol consumption may mitigate the beneficial effects of weight loss and support the need for larger Randomized Controlled Trials (RCTs) to better investigate the metabolic effects of moderate alcohol intake in humans.

## 1. Introduction

Alcohol (i.e., ethanol) is one of the most widely used recreational substances by humans and is consumed regularly by much of the population. Recent data reported that as many as ~58% of adults consumed alcohol within the previous month [1]. Other evidence from the National Health and Nutrition Examination Survey (NHANES) suggests that alcohol constitutes an estimated 5% of total daily energy intake in U.S. adults [2]. Despite the widespread consumption of alcohol, the health effects associated with its use have not been firmly established.

While the link between excessive alcohol intake and poor health outcomes, including liver cirrhosis, obesity-related cardiometabolic disorders, and mortality, is well-documented in humans, the consequences of moderate alcohol consumption remain obscure. Experimental intervention studies to examine the effects on body weight gain and obesity have been scarce and largely inconsistent, reporting either a positive association [3,4] or no effect [3,4,5,6,7] with alcohol intake. Routine alcohol consumption has also been associated with adipose tissue (AT) distribution, with limited cross-sectional studies describing a positive correlation with increased visceral adipose tissue (VAT) accumulation [8,9,10,11]. Yet, other studies suggest either an inverse or no association [7,12]. Interestingly, moderate alcohol consumption has been suggested to have both beneficial and unfavorable effects on cardiovascular morbidities and insulin sensitivity [13].

Many of the discrepancies in prior studies are due to the inclusion of various alcohol types (i.e., wine, beer, and spirits), which prevents the characterization of ethanol-specific effects, as wine and beer contain antioxidants and other micronutrients. The interpretation of existing data is also complicated by different intervention time periods, potential sex differences, the use of self-reported dietary data and other potential confounders. Many of these analyses have been primarily epidemiological and cross-sectional in nature; hence, there is a paucity of data from longitudinal assessments in humans to examine the metabolic response to routine moderate alcohol consumption in randomized controlled trials (RCTs). In addition, although ethanol consumption has been shown to be a risk factor for health consequences associated with weight gain, no study has examined the physiological effects of alcohol intake during a weight loss intervention, despite the favorable effects of energy restriction.

We report, herein, the first pilot RCT to investigate the effects of moderate alcohol consumption on metabolic outcomes during weight loss in women with obesity who consumed 30% energy restricted diets and were randomized to either an ethanol-free control (CTL) group or a group that consumed 35 g ethanol daily (EtOH) for eight weeks. We hypothesized that alcohol consumption would mitigate the favorable metabolic health effects resulting from weight loss.

## 2. Materials and Methods

### 2.1. Participant Characteristics

The study reported herein was conducted according to the guidelines in the Declaration of Helsinki. All participants were given verbal and written explanations about the study, provided signed informed consent, and received a monetary stipend. The study was approved by the Association for the Accreditation of Human Research Protection Programs (AAHRPP) accredited Pennington Biomedical Institutional Review Board. The study was registered at ClinicalTrials.gov (NCT 03521817).

Participants were healthy, pre-menopausal females who were 21–40 years of age with a BMI between 27–50 kg/m^2^. Twelve participants were enrolled in the study. The inclusion criteria are as follows: (1) being willing to practice appropriate birth control, (2) being willing to eat at Pennington Biomedical Research Center (PBRC) at least 3 times per week, (3) beingwilling to consume alcohol (EtOH group), (4) being willing to abstain from alcohol (CTL group), and (5) being a daily or almost daily drinker, defined as typically consuming at least 8 drinks per week, but no more than 4 per day. Exclusion criteria included but are not limited to: (1) non-drinkers of alcohol, (2) habitual binge drinkers, defined by the consumption of ≥ 4 standard drinks per day or ≥ 28 drinks per week, (3) self-reported alcoholics or a history of alcoholism, (4) any attendance or inpatient stay for alcohol or drug treatment, (5) display any characteristic of current or future substance abuse disorders, (6) presence of any psychiatric, behavioral, or medical disorder that, in the opinion of the PIs, Co-Is, or MI, may interfere with study participation, the ability to adhere to the protocol, or has the potential for increased substance abuse, (7) prescription medications that interact with alcohol intake, (8) abnormal screening laboratory safety tests, (9) smokers, (10) diagnosis of Type 1 or 2 diabetes mellitus, cancer, or major organ disease, (11) serious digestive disorders, (12) conditions that affect metabolism or body weight (i.e., uncontrolled thyroid conditions, bariatric surgery, pregnancy, breastfeeding), (13) hysterectomy and/or hysterectomy with bilateral salphingo-oopherectomy, (14) hormonal pharmaceutical contraceptives including oral contraception (birth control pills), injectables (Depo-Provera), or the patch (Xulane), (15) Polycystic Ovary Syndrome (PCOS), and (16) use of medications that affect body weight or metabolism (i.e., atypical antipsychotics, weight loss medications).

### 2.2. Study Design

This study was a single blind between-subject randomized controlled study design. The investigators were blinded to the treatment group, but due to the nature of the study, participants were not blinded. All women underwent a 30% energy restriction for 8-weeks and were randomized to either an ethanol-consuming group or a non-ethanol control group at the start of the study. Participants initially visited the center for a screening visit (SV). If they volunteered and met the inclusion/exclusion criteria, they attended a pre-randomization visit. Following the pre-randomization visit, an alcohol tolerance test occurred (with study dose). Pending acceptance and adherence to the alcohol tolerance test, participants returned to the center for Clinic Visit (CV) 1 and randomization. The intervention lasted 8 weeks. Participants were required to come to the center a minimum of 3 times per week. Finally, participants came in for the final CV. Thus, participation lasted approximately 10–12 weeks in total.

#### 2.2.1. Screening Visit 1

SV was conducted the morning after a ~10-h overnight fast. Demographics, vital signs, and medical history were assessed. A physical exam was administered. Anthropometric characteristics (i.e., height, metabolic weight, body mass index (BMI), blood pressure, and waist and hip circumference) were measured. A blood sample was collected and analyzed for laboratory safety tests. Menstrual cycle status was determined.

A semi-standardized Lifestyle Interview was administered by trained behavioral staff (Master’s Level Psychologists) and overseen by a clinical psychologist (CKM). The interview was used to identify barriers to participation in the study, including scheduling challenges, ability to attend appointments, etc. The Substance Abuse Subtle Screening Inventory (SASSI) [14,15] and Michigan Alcoholism Screening Test (MAST) [16,17] questionnaires were administered. All participants were also screened and excluded based on the presences of psychological conditions that could interfere with the ability to adhere to protocol or indicate that participating in the research could be unsafe, for example, due to a history of substance dependence. Specifically, the Structured Clinical Interview for DSM-5 Clinical Version (SCID-5-CV [18]) was administered to identify mood disorders, psychotic disorders, substance abuse, etc. Furthermore, the Structured Clinical Interview for DSM-5 Personality Disorders (SCID-5 PD [19]) was administered to identify DSM-5 Personality Disorders.

This, and other information, was used to provide an overall eligibility assessment for each candidate. A multidisciplinary team consisting of behavioral experts, dietitians, clinical staff, a Medical Investigator (FLG), and study staff discussed and approved candidates for official admission to the study.

#### 2.2.2. Pre-Randomization Visit

Before enrollment in the study, all women were required to undergo an overnight alcohol tolerance test at the required ethanol dose. This was the dose of alcohol that was to be consumed for the duration of the study in the EtOH group. Furthermore, women were provided with a step log (pedometer provided; New Lifestyles YAMX Digi-Walker) and asked to return it at CV1. Pending adherence to the overnight alcohol test, a one-week ethanol-free washout following the overnight tolerance test, and the return of the pedometer with step count, participants underwent CV1.

The purpose of the ethanol-free washout following the overnight tolerance test was two-fold. First, participants demonstrated the ability to forgo alcohol for 1 week. Secondly it provided a stable baseline period for all subjects prior to study enrollment at CV1.

#### 2.2.3. Clinic Visit (CV) 1

At CV1 and 2, women reported to PBRC after an overnight fast during the luteal phase of the menstrual cycle. Anthropometric characteristics (height; metabolic weight; mean arterial pressure (1/3 [systolic BP − diastolic BP] + diastolic BP)) were measured.

Subcutaneous abdominal AT (SAT) and visceral AT (VAT) volumes were defined and quantified with magnetic resonance imaging (MRI) using a 3.0 T scanner (GE, Discovery 750 w) by obtaining ~581 images from the dome of the liver to the pubic symphysis. Images were analyzed by a single trained analyst. Estimates of VAT and SAT volumes were converted to mass using an assumed density of 0.92 kg/L. Blood draws were performed, and serum and plasma were archived.

### 2.3. Randomization

Eligible participants were then randomized (1:1) to consume 35 g/day of ethanol (EtOH group; *n* = 7) or to control (CTL; *n* = 5) via block randomization (using SAS 9.4) for 8 weeks. After randomization, participants began the dietary intervention.

### 2.4. 8-Week Dietary Intervention

Participants were provided all meals. Meals were prepared by the PBRC metabolic kitchen (5-day meal rotation). Participants were given both verbal and written instructions as to the consumption of the food. On weekdays, a minimum of one meal (i.e., breakfast, lunch, or dinner) was consumed at PBRC at least 3 times per week, (excluding holidays), whereas the other weekday meals were packaged for takeout. In addition, alcohol, weekend, and holiday meals were packaged for takeout. Participants’ energy requirements were determined from their estimated resting metabolic rate (RMR), calculated via the Mifflin-St. Jeor formula [20] with an activity factor of 1.5. The energy requirements were then multiplied by 0.70 so that all participants will restrict energy needs by 30% compared to weight maintenance. Each group (control and ethanol) had the same 30% reduction in calories and will consume the same percentage of each additional macronutrient (20% protein, 50% carbohydrate, and 30% fat).

The ethanol group consumed a 30% energy restriction diet that also included ~2.5 standard drinks, or 35 g of ethanol, administered as 80-proof distilled spirits (e.g., 80 proof gin, rum, vodka, whiskey, or tequila). In the United States, one “standard” drink contains roughly 14 g of pure alcohol. The remaining calories (Mifflin-St. Jeor formula, with an activity factor of 1.5 multiplied by 0.70 minus the ~240 kcals from ethanol) was consumed as 20% protein, 50% carbohydrate, and 30% fat. The control group l consumed these ~240 kcal as included in the aforementioned macronutrient percentages (20% protein, 50% carbohydrate, and 30% fat) as a beverage to match the ethanol group.

The alcohol was purchased, logged, handled, and dispensed by the study pharmacist. Measured doses of alcohol were prepared by the study pharmacist. Sealed, individual bottles (~7) of alcohol were dispensed to the participant once per week (on the Monday; excluding holidays) through the outpatient clinic. Each bottle contained a pre-measured dose of alcohol, and participants drank the pre-measured dose of alcohol each day of the week.

Participants were asked to have an in-clinic check in once per week when coming for their in-house meal (generally Monday am). At this check a metabolic weight was obtained, adverse events and changes in medication were assessed. The ethanol group was also administered the modified weekly version of the alcohol-related questionnaires (SASSI and MAST).

### 2.5. Instructions for Alcohol Consumption

Participants were given general information about alcohol and informed of the effects of its use on the body. The participants were warned of the risks associated with performing daily activities and operating vehicles or other machinery after alcohol ingestion. Alcohol was instructed to be consumed when participants were in for the evening and driving was concluded for the day. Subjects were provided information about the warning signs of alcohol dependency.

### 2.6. Dietary and Alcohol Compliance

Subjects were instructed to report if any additional food, beverages, and alcoholic drinks were consumed. Subjects who were under-compliant (<85%) or over-compliant (>100%) were counseled by study staff on the importance of compliance.

### 2.7. Participant Follow-up 

At the end of the intervention, participants were contacted (phone) by trained study staff at 2 weeks and 4 weeks after completion of the study in order to assess if the women have acquired any alcohol abuse and/or dependency issues.

### 2.8. Blood Parameters

A chemistry panel was performed at SV1, CV1, and CV2. This included glucose, cholesterol (HDL and LDL), triglyceride, and uric acid. Furthermore, insulin, free fatty acids (FFA), and glycerol (GLY) were measured with standardized procedures.

### 2.9. Statistical Analysis

Analyses were carried out using SAS, Version 9.4 (SAS Institute, Cary, NC) with a significance level of α = 0.05. All data reported are mean ± SEM. Two-way ANOVAs were used to test if changes in covariates (Week 8 — Baseline) differed between treatment groups (primary outcome). Treatment effects, which represent the change induced in the EtOH group relative to the change in the CTL group, are denoted with symbol Δ. Normality of the residuals from the mixed model were checked and observations with a residual value of ± 3 were investigated. One participant in the CTL group was dropped from the study due to time commitments, and another in the CTL group was excluded from the analysis, as she was non-compliant with the study design.

## 3. Results

The analyses included 10 women who completed the study (CTL group—*n* = 3; EtOH group—*n* = 7) who were of Caucasian (*n* = 6), African American (*n* = 3), or Other decent (*n* = 1). One person (Other) was of Hispanic, Latino, or Spanish Origin. At SV1, they were 33 ± 3 years with a mean body weight of 97.8 ± 4.4 kg and BMI of 35.4 ± 1.6 kg/m^2^. The control group had 4917 ± 1706 step/day and the EtOH group had 6902 ± 1117 steps/day at baseline (*p* = 0.36). The clinical and metabolic characteristics of participants in the CTL and EtOH groups are included in Table 1.

There was no significant difference in the change in body weight between the CTL and the EtOH groups (Δ = −1.2 ± 1.4 kg; *p* = 0.43). There was no significant difference in the change in SAT (1A; Δ = 0.05 ± 0.3 kg; *p* = 0.87) or VAT (1B; Δ = −0.18 ± 0.14 kg; *p* = 0.23) mass between the CTL and the EtOH groups. Furthermore, there was no significant difference in the change in triglycerides (2A; Δ = −42.9 ± 24.3 mg/dL; *p* = 0.11) or cholesterol (2B; Δ = −21.1 ± 11.8 mg/dL; *p* = 0.11) between the CTL and the EtOH groups. The decrease in mean arterial pressure was significantly attenuated by alcohol consumption in the EtOH group as compared to the CTL group (Δ = −8.0 ± 2.8 mmHg; *p* = 0.02). There was no significant difference in the change in mean steps between the CTL and the EtOH groups (Δ = 743 ± 1547 steps/d CTL, Δ = −113 ± 1013 steps/d EtOH, Δ = 855.8 ± 1849.3 steps/d; *p* = 0.66).

## 4. Discussion

The authors believe this to be the first study to test the effects of EtOH consumption on the cardiometabolic effects of weight loss in premenopausal women with obesity during controlled feeding. The results from this pilot study suggest that the ingestion of EtOH may have negative effects on the metabolic benefits of weight loss. Our findings demonstrate that the decrease in mean arterial pressure was attenuated in the EtOH group, relative to the CTL group. In addition, decreases in other outcomes, including VAT and circulating lipids (triglycerides and cholesterol), tended to be attenuated with alcohol consumption. With the increasing focus on personalized medicine, alcohol consumption may play a significant role as a modifiable risk factor affecting the metabolic health outcomes associated with weight loss. Future work may consider adding physical activity to determine if it can improve metabolic health outcomes with alcohol consumption.

Limited randomized trials have examined the effects of EtOH on circulating lipid levels. A previous controlled feeding study recruited weight-stable premenopausal women who were ~80–130% of desirable weight and were provided 30 g of alcohol per day for 60 days [21]. Alcohol consumption did not affect total cholesterol or triglyceride levels, but HDL increased, and LDL decreased [21]. Another crossover-controlled feeding study examined postmenopausal women who were 90–140% of ideal in weight maintenance [22]. This study performed a dose response with three treatment groups including control, 15 g alcohol, and 30 g alcohol. The 15 and 30 g alcohol groups had decreased triglycerides compared to the control group. The 30 g alcohol group decreased total cholesterol compared to the control group, and the 15 g alcohol group was not different than either group. The 30 g alcohol group increased HDL compared to control and 15 g alcohol drink group, whereas LDL was decreased in the 15 and 30 g alcohol groups compared to the control group [22]. Similarly, a study in women with obesity was randomized to either ~2.5 drinks of white wine or grape juice [23]. Post study, the white wine group had decreased triglycerides and LDL cholesterol and increased HDL cholesterol. Herein, we found that HDL levels were fairly consistent between groups, but triglycerides tended to have a greater reduction in the EtOH vs. the control group. Overall, lipid metabolism is likely affected by moderate ethanol intake. In energy balance (weight maintenance) alcohol may have positive health effects on lipid metabolism, whereas alcohol may attenuate the positive effects of weight loss on lipid metabolism in women.

Interestingly, early data from Eric Jequier’s group demonstrated that the addition of alcohol to the diet decreased lipid (fat) oxidation, suggesting that ethanol consumption may favor lipid storage [24] in a cross-sectional study with low to modest habitual alcohol intake. This coincides with our results suggesting that VAT loss tended to be attenuated in the EtOH vs. control groups. However, the effects of ethanol on adipose tissue (AT) distribution are controversial. Several studies have reported that routine alcohol intake is positively correlated with increased visceral adipose tissue (VAT) accumulation [8,9,10,11]. Other studies suggest either an inverse or no association with central adiposity [7,12]. A previous study examined the effects of drinking red wine (~17 g ethanol) over two years and found no differences between the ethanol groups and control group in visceral adiposity [25]. The results reported herein found that VAT loss tended to be attenuated with EtOH consumption. A strong positive association between visceral adiposity and blood pressure exists [26]. Interestingly, to date, alcohol intake has been shown to have somewhat mixed effects on blood pressure response [27,28,29], however, and not surprisingly the current study found a strong attenuation in mean arterial blood pressure loss with EtOH.

It is possible that alcohol consumption may have diuretic action and influence dehydration; however, the extent of these effects is not fully understood. A previous RCT study showed that spirits (consumed at an amount similar to our study) caused only a small and transient diuretic effect at 4 h, and that there were no differences in 24 hr urine output or urine osmolarity when compared to subjects that consumed water [30]. Another study reported that more urine was excreted after 4 h when beer (4% alcohol), compared to the non-alcoholic beverage, was consumed during euhydration (*p* < 0.001), while there was no significant difference between groups during hypohydration (*p* = 0.06) [31]. Yet, a subsequent RCT using the beverage hydration index found that cumulative urine output at 4 h after ingestion of lager beer (4% alcohol) was not different from the response to water ingestion [32]. Taken together, these data suggest that the moderate consumption of alcohol may play a limited role in hydration. Of note, in our study, the alcoholic drinks were consumed with non-caloric mixers, which contain water.

Furthermore, various hormones, including vasopressin, can impact hydration and influence metabolic outcomes such as blood pressure, and studies have shown that alcohol consumption can alter vasopressin [33]. Our data demonstrate that alcohol consumption attenuated the lowering of mean blood pressure during weight loss, relative to the control group; however, neither vasopressin, nor other similar hormones, were measured in our study. Though hormonal regulation is plausible, the precise mechanisms underlying these findings need to be elucidated in future studies, as it is likely that there are several independent mechanisms acting to increase blood pressure with alcohol consumption [34].

In previous studies, moderate alcohol consumption has been associated with benefits to glycemia particularly in nondiabetic subjects [35,36,37,38]. Postmenopausal women with 90–140% of the ideal weight (27.4 kg/m^2^) were enrolled in a randomized crossover trial with 0, 15, or 30 g of alcohol [39]. The study found that fasting insulin decreased in the 30 g of alcohol vs. control conditions. Insulin sensitivity improved with the consumption of 30 g of alcohol vs. control conditions in non-diabetic premenopausal women [39]. Similarly, in a study where women with overweight were randomized to either white wine or white grape juice for six weeks, fasting insulin was lower in the white wine vs. grape juice groups [23]. Alcohol seems to confer benefits to insulin sensitivity during weight maintenance. However, in a nonrandomized study that provided 30 g of alcohol to insulin resistant individuals, no benefits to insulin sensitivity were seen [40]. In the current study, our results suggest no difference between groups. This may be due to the difference in baseline body weight of the participants among studies, or the fact that, in energy balance (weight maintenance), alcohol may have positive health effects on insulin sensitivity, whereas alcohol may attenuate the positive effects of weight loss on insulin sensitivity in women.

Well-powered longitudinal assessments from a randomized, controlled trial (RCT) with clinically relevant end points are necessary in order to examine the influence of chronic alcohol consumption on cardiometabolic health, central adiposity, and the mechanisms underlying ethanol-associated changes in metabolism. Post-hoc power analyses indicated that if 38 total participants were enrolled (*n* = 19/group), this study would have been powered to see differences in all of the main variables that did not reach significance: visceral adipose tissue, circulating lipids (triglycerides and cholesterol), and uric acid. NHANES data underestimates the overall energy intake due to self-report methodology and further underestimates the percentage daily alcohol energy intake in alcohol consumers. In persons that consume alcohol, energy intake from ethanol is estimated to be ~10% [41]. Therefore, if the average daily total energy intake is ~2400 kcal in women with obesity, most alcohol users are likely consuming ~240 kcal/d (10% of total energy) from alcohol. Hence, our dose of ethanol is externally valid.

This study has numerous strengths. These include that it was a randomized controlled trial (RCT), and a controlled feeding trial. It is important to note that all food (and beverage) intake was provided for all subjects, and the intervention did not affect physical activity. For eight weeks, participants were only provided with, and expected, food and beverage from the Pennington Biomedical metabolic research kitchen. The study did not involve behavior change (i.e., behavioral weight loss), nor was it recommended. As expected, physical activity did not change during the course of the intervention. While a pilot study, multiple effect sizes were robust. Some weaknesses include the lack of physical activity data throughout the study, no measurement of hydration status, low sample size, and the lack of a true control group. Furthermore, only premenopausal women were included—given that there are sex differences in adipose tissue distribution [42] (a primary endpoint) and likely the mechanisms that influence this and other metabolic outcomes—in order to reduce potential confounders.

## 5. Conclusions

These pilot data provide potential evidence that moderate alcohol consumption may counteract the beneficial effects of weight loss and support the need for larger RCTs to better investigate the metabolic effects of moderate alcohol intake during weight loss in humans. Personalized medicine remains a focus of the National Institutes of Health (NIH). While preliminary, this pilot study suggests that a dietary pattern which incorporates moderate alcohol consumption may attenuate some of the beneficial effects of weight loss, but well-powered follow-up studies are needed.

## Figures and Tables

**Table 1:** Baseline, treatment, and changes in the clinical and metabolic characteristics of participants in the ethanol-free control (CTL) and the group that consumed 35 g ethanol daily for eight weeks (EtOH).

	CTL Δ W0 vs. W8	EtOH Δ W0 vs. W8	Δ CTL vs. Δ EtOH	Effect Size Δ CTL vs. Δ EtOH	*p*-Value Δ CTL vs. Δ EtOH
BMI (kg/m^2^)	−2.2 ± 0.4 ^a^	−2.0 ± 0.3 ^a^	−0.2 ± 0.5	−0.2	0.76
Body weight (kg)	−6.6 ± 1.2 ^a^	−5.4 ± 0.8 ^a^	−1.2 ± 1.4	−0.6	0.43
SAT (kg)	−1.1 ± 0.3 ^a^	−1.2 ± 0.2 ^a^	0.05 ± 0.3	0.1	0.87
VAT (kg)	−0.2 ± 0.1	−0.1 ± 0.1	−0.2 ± 0.1	−0.9	0.23
Triglycerides (mg/dL)	−26.3 ± 20.3	16.6 ± 13.3	−42.9 ± 24.3	−1.2	0.11
FFA (mmol/L)	0.1 ± 0.1	0.2 ± 0.1 ^a^	−0.1 ± 0.1	−0.3	0.62
Glycerol (mmol/L)	−0.01 ± 0.02	0.02 ± 0.02	−0.03 ± 0.03	−0.8	0.33
Cholesterol (mg/dL)	−26.7 ± 9.9 ^a^	-5.6 ± 6.5	−21.1 ± 11.8	−1.2	0.11
HDL (mg/dL)	−5.7 ± 3.7	-4.3 ± 2.4	−1.4 ± 4.4	−0.2	0.76
LDL (mg/dL)	−15.7 ± 8.3	-4.6 ± 5.4	−11.1 ± 9.9	−0.8	0.29
Mean Arterial Pressure (mmHg)	−8.3 ± 2.3 ^a^	−0.4 ± 1.5	−8.0 ± 2.8	−2.0	0.02
Glucose (mg/dL)	−3.0 ± 4.5	3.3 ± 3.0	−6.3 ± 5.4	−0.8	0.28
Insulin (mU/L)	−6.8 ± 3.3	−5.2 ± 2.1 ^a^	−1.6 ± 3.9	−0.3	0.70
HOMA-IR	−1.7 ± 0.85	−1.1 ± 0.6	−0.6 ± 1.0	−0.4	0.58
Uric Acid (mg/dL)	−0.5 ± 0.3	0.3 ± 0.2	−0.8 ± 0.4	−1.4	0.07

Values presented as mean ± SEM; ^a^ *p* < 0.05 for within-group change in measured outcome
